# Miltefosine for the treatment of cutaneous leishmaniasis—A pilot study from Ethiopia

**DOI:** 10.1371/journal.pntd.0009460

**Published:** 2021-05-28

**Authors:** Saskia van Henten, Annisa Befekadu Tesfaye, Seid Getahun Abdela, Feleke Tilahun, Helina Fikre, Jozefien Buyze, Mekibib Kassa, Lieselotte Cnops, Myrthe Pareyn, Rezika Mohammed, Florian Vogt, Ermias Diro, Johan van Griensven

**Affiliations:** 1 Department of Clinical Sciences, Institute of Tropical Medicine, Antwerp, Belgium; 2 Department of Dermatology, University of Gondar Hospital, Gondar, Ethiopia; 3 Department of Internal Medicine, College of Medicine and Health Sciences, Wollo University, Dessie, Ethiopia; 4 Boru Meda Hospital, Dessie, Ethiopia; 5 Leishmania Research and Treatment Center, University of Gondar Hospital, Gondar, Ethiopia; 6 National Centre for Epidemiology and Population Health, Research School of Population Health, College of Health and Medicine, Australian National University, Canberra, Australia; 7 The Kirby Institute, University of New South Wales, Sydney, Australia; Brighton and Sussex University Hospitals NHS Trust, UNITED KINGDOM

## Abstract

**Background:**

Cutaneous leishmaniasis (CL) in Ethiopia, caused by *Leishmania aethiopica*, is often severe and hard to treat compared to CL caused by other species elsewhere. Miltefosine is the only oral anti-leishmanial drug, with a favorable side-effect profile compared to routinely available sodium stibogluconate (SSG), but evidence about its use for *L*. *aethiopica* is lacking.

**Methodology and principal findings:**

In an observational cohort study, treatment outcomes, safety and adherence among CL patients who required systemic treatment and received miltefosine for 28 days in Boru Meda Hospital and University of Gondar Hospital were studied. Patient cure was defined as 100% flattening for non-ulcerated lesions and 100% flattening and 100% re-epithelization for ulcerated lesions. Outcomes were documented for day 28, 90 and 180, both per site, and pooled, adjusting for site as a fixed effect with effect coding. Among 94 included patients (32 in Gondar, 62 in Boru Meda), median lesion duration was 12 months, median size six cm, and mucosal involvement (46.8%) and diffuse (30.9%) lesions were common. Adherence to miltefosine was good, and side-effects were tolerable. Initial outcomes at day 28 were promising, with 68.8% and 94.0% of patients having good improvement or cure in Gondar and Boru Meda respectively. In Boru Meda, outcomes were good with 72.7% and 72.9% cure at day 90 and day 180 respectively. In Gondar, results were less promising, with only 12.5% and 26.7% cure at day 90 and day 180, although confidence intervals were wide. In pooled estimates, 48.7% of patients reached cure at day 180, and 32.3% relapsed. Outcomes were better in Boru Meda Hospital, for smaller lesions and for mucosal lesions.

**Conclusions/Significance:**

Based on miltefosine’s good initial response, tolerable side-effects, tablet-form, we propose to include miltefosine for future clinical trials using extended treatment schedules, combination therapy, or targeting specific subgroups.

**Trial registration:**

ClinicalTrials.gov NCT04004754.

## Author summary

Cutaneous leishmaniasis (CL) in Ethiopia is caused by *Leishmania aethiopica*, resulting in relatively severe lesions, which are hard to treat. Currently, most patients are treated with pentavalent antimonials, although effectiveness seems poor, with many patients requiring repeated treatment cycles before good response is seen. Miltefosine is the only oral drug available for leishmaniasis treatment and has a good safety profile compared to other treatment options. Although evidence on the use of miltefosine for CL in other regions looks promising, reports from Ethiopia are lacking.

We systematically recorded outcomes and side-effects for patients with CL lesions that required systemic treatment and who were treated with 28 days of miltefosine in a prospective study in two hospitals in Ethiopia. Side-effects were common but generally mild. Although all patients showed improvement after 28 days, overall, around half of patients were cured after six months, with relapse being common. Therefore, we propose to include miltefosine in future clinical trials, but to adapt the treatment regimen using combination therapy or treatment extension to improve overall outcomes and reduce relapse.

## Introduction

Cutaneous leishmaniasis (CL) is transmitted by the bite of *Leishmania* infected sandflies, affecting between 0.7 and 1.2 million people annually in over 90 countries worldwide [1]. Although not life-threatening, it severely impacts lives through skin mutilation and stigma. There are three CL forms; localized CL (LCL), which is limited in size and has higher chance of self-healing; mucocutaneous leishmaniasis (MCL) which affects the mucosa; and diffuse CL (DCL) with multiple or diffuse lesions [2] and poor response to treatment [3]. Systemic treatment is required for MCL, DCL and severe (large, non-healing or on lesions sites unsuitable for local therapy) forms of LCL [4].

In Ethiopia, CL is estimated to affect between 20,000 and 40,000 persons per year [1,3], with over 28 million people at risk of infection [5]. Compared to CL caused by other species, CL lesions caused by *L*. *aethiopica* are more severe and diverse in clinical presentation [6]. In contrast to most countries where CL is characterized by ulcers or nodules, plaques, crusting, and erythema are more common in Ethiopia [7]. Most cases occur in remote rural areas with limited access to healthcare. Although species typing is not routinely done in clinical practice, all clinical CL samples typed to date in Ethiopia were due to *L*. *aethiopica*, with only a single exception of *L*. *tropica* [8]. CL caused by *L*. *aethiopica* is considered to be a zoonotic disease, with hyraxes serving as animal reservoirs, and transmission occurring via sandfly bites of *Phlebotomus pedifer* and *P*. *longipes* [9,10].

Four *Leishmania* drugs are mentioned for systemic treatment of CL in the Ethiopian National Guideline [11]: pentavalent antimonials (Sodium stibogluconate (SSG) or meglumine antimoniate); paromomycin; miltefosine and liposomal amphotericin B (AmBisome). All options are recommended equally without preference for one particular regimen, due to lack of evidence from high-quality studies. Not one clinical trial with these drugs has been carried out in Ethiopia to date.

Due to general scarcity of medication and prioritization of visceral leishmaniasis (VL) treatment in Ethiopia, only limited treatment options are routinely available for CL. Intramuscular injection of SSG is the most commonly used treatment [12,13], but it requires daily painful injections and can have dangerous side-effects, such as cardiotoxicity, pancreatitis and renal toxicity [11].

Miltefosine is the only oral drug available for leishmaniasis treatment, which opens the possibility for outpatient treatment and hence scale-up of care. Furthermore, its side effect profile is low in comparison with other treatment options, gastro-gastrointestinal upset being the most common [14]. Although miltefosine is supplied for combination treatment of VL cases by some non-governmental organizations, it is not routinely available for CL treatment in Ethiopia.

Several clinical trials have investigated the use of miltefosine, but so far, they have been limited to Latin America and Iran with some case reports from Europe [15]. Most studies show that miltefosine is superior or non-inferior to antimonials in terms of efficacy [16–19], although it seems miltefosine effectiveness differs per species and region [20,21]. *In vitro* studies have shown high susceptibility of *L*. *aethiopica* to miltefosine [22], but outcomes of patients receiving miltefosine have never been documented systematically.

Therefore, evidence on miltefosine for the treatment of CL caused by *L*. *aethiopica* is urgently needed. We conducted this study, embedded in routine care in a CL-endemic setting in Northern Ethiopia, to establish whether using miltefosine is effective and safe for CL. Findings from this study will be crucial to determine whether miltefosine should be evaluated further in clinical trials.

## Methods

### Ethics statement

This study was approved by the ethical review committees of the Institute of Tropical Medicine in Antwerp, the University Hospital of Antwerp, the University of Gondar, and the National Ethiopian ethical review board. Written informed consent was obtained from all participants (or from the parent/guardian for children), with additional written assent collected from children aged 12–18. This study is registered at ClinicalTrials.gov as NCT04004754.

### Study setting

This study was conducted in two different sites in North-West Ethiopia, which serve as referral centers for CL patients; University of Gondar Hospital in the North-West and Boru Meda Hospital in the East of the Amhara region.

At the University of Gondar Hospital, cutaneous leishmaniasis (CL) care is shared by the Department of Dermatology and the Leishmaniasis Research and Treatment Center (LRTC–supported by the Drugs for Neglected Diseases initiative (DND*i*)), which has experience with Good Clinical Practice compliant drug trials on VL treatment. Most patients with skin conditions first visit the dermatology clinic. In case CL is suspected, patients are sent to the LRTC for skin slit smear microscopy with Giemsa staining for parasitological examination. The results are then sent to the LRTC physician for treatment decision which may also be empiric if microscopy results are negative but the clinical picture is clear. At the LRTC, the routine treatment option is SSG. Patients with less severe lesions may be sent back to the dermatology clinic for cryotherapy or intralesional injection with SSG. Organ function tests are routinely done at the LRTC for patients prior to starting systemic treatment. Because beds in the LRTC ward are limited, most CL patients receive SSG as an outpatient, with drug administration done at the district hospital level.

Boru Meda Hospital serves as a general hospital with specialized services in dermatology and ophthalmology. It has a dedicated dermatology clinic with a high caseload of leprosy and CL. It has a dermatology ward with 40 beds. CL suspected patients are routinely screened by skin slit smear microscopy. Patients requiring systemic treatment mostly receive SSG with allopurinol and are admitted for treatment, while cryotherapy and intralesional SSG is given for smaller lesions. Organ function tests are not routinely available.

Antileishmanial medicines are made available in Ethiopia with support of international organizations such as Medicines sans Frontières, the World Health Organization (WHO) and DND*i*. Miltefosine is not routinely available in either of the two settings for CL treatment (although frequently available in the LRTC for VL patients, and occasionally used for CL cases that are difficult to treat). There was a recent miltefosine donation within the Institute of Tropical Medicine (Antwerp)—University of Gondar collaboration agreement, which was distributed among the two sites based on the expected caseload.

### Study design, population and recruitment

This was an observational cohort study among patients with CL who received miltefosine following routine clinical care decisions. Patients with a clinical or parasitological diagnosis of CL who were planned to be started on miltefosine were consecutively invited to participate in the study. Since the decision to administer miltefosine or not was part of routine clinical care, there were no specific exclusion criteria for this study. However, physicians were aware that women of child-bearing age should not receive miltefosine when pregnant or breastfeeding and strongly advised all other women the use of contraception during and up to four months after use of miltefosine. Miltefosine was only given to patients who required systemic treatment, as local treatment options were available during the study period.

Because of the exploratory nature of this study no formal sample size calculation was done. The study recruitment period was May—November 2019, and patients were followed until June 2020.

### CL confirmation

CL diagnosis followed routine procedures of clinical consideration, slit skin smear (SSS) and microscopic examination of the Giemsa stains. Additionally, for study purpose, a real-time PCR targeting the kinetoplast DNA (kDNA) minicircles [23] was done on the microscopy slides for molecular parasitological confirmation and species typing was performed on five selected samples by sequencing the ITS-1 gene fragment [24] (see S1 Text for detailed methods).

### Miltefosine treatment and follow-up

Patients were given a standard course of 28 days of miltefosine treatment [11]. Patients above 45 kg received 150 mg/day, patients 30–44 kg received 100 mg/day, while children below 30 kg received allometric dosing according to *Mbui et al*.[25].

In Gondar, patients were not admitted, but received the complete course of miltefosine for ambulatory use after instructions on usage and potential side-effects. In Boru Meda Hospital, patients started on miltefosine were hospitalized for one week, in which nurses actively administered drugs daily and monitored for side-effects. Patients were then discharged with counselling how to continue home-based treatment for the 21 remaining days.

Patients were asked to return on day 28 (window 23–39), day 90 (window 60–120), and day 180 (window 120–240 days) of starting treatment. Additionally, they were requested to come back between day 28 and day 90 in case the lesion was not responding or worsening. Patients were called in case they were late for their planned visit.

### Data collection and analysis

A paper data collection form was used to collect socio-demographic, history, and physical assessment data. Data quality was checked and afterwards entered into a database using REDCap.

#### Analysis populations

Three different analysis populations were used: Per Protocol (PP), Intention to Treat (ITT) and an analysis using only confirmed CL patients; with PP as the main analysis and ITT and confirmed cases only as sensitivity analyses. For the PP analysis, only patients who came within the time-windows of the respective visits were considered. Additionally, patients that were given a treatment extension or alternative treatments before outcome ascertainment, patients who took miltefosine for less than 23 days, and patients with a treatment interruption of more than one week were excluded. For the ITT analysis, all patients started on miltefosine were included, with patients who came outside of the study visit window or who did not come at all recorded as having no response (worst-case scenario).

#### Definitions

To assess treatment outcomes at different time-points, the proportion of the lesion area that flattened and showed re-epithelization was used compared to the first study visit. In case of multiple lesions, the worst lesion determined the patients’ outcome category, meaning patients were only considered cured if all lesions were cured. We adapted internationally used criteria and cutoffs for outcome definitions [26,27]: Cure was defined using 100% reepithelization and/or flattening according to international recommendation [26], good response as 50–99% reepithelization and/or flattening, partial response as 1–49% reepithelization and/or flattening, no response as 0% reepithelization or flattening, and relapse as worsening of lesions after initial response, or appearance of new lesions on the same site after previous improvement (Table 1). The physician’s assessment of flattening and/or reepithelization was used, aided by photographs which were taken during every study visit.

**Table 1:** Definitions of the different outcome categories.

	Ulceration present	No ulceration present
**Cure**	100% re-epithelization and 100% flattening	100% flattening
**Good response**	50–99% re-epithelization and/or 50–99% flattening^a^	50–99% flattening
**Poor response**	1–49% re-epithelization and/or 1–49% flattening^a^	1–49% flattening
**No response**	0% reepithelization or 0% flattening	0% flattening
**Relapse**	New lesion on the same site or worsening of previously improving lesion	

^a^The worst response is binding, so i.e. if a patient has an ulcerated lesion that has completely flattened but reepithelization of approximately 80% has occurred, the lesion will be considered to have good response.

Failure was defined as no response at day 28; no response, partial response, or relapse at day 90; and good response, partial response, no response and relapse at day 180. Additional details of information on outcome definitions is defined in S1 Text.

Adherence was measured as the number of days missed on the adherence checklist given to the patients and the self-reported adherence. It both were available, the average was used, if only one was available this was used alone. Two different approaches were used; one in which patients without adherence information were excluded, and one in which patients without adherence information were interpreted as having missed ten days of treatment (conservative approach). If patients missed their day 28 visit, adherence information was obtained either on subsequent visits, or over the phone.

We used the physician’s interpretation of the lesion for the classification of CL, as no clear definitions of CL types in Ethiopia exist. In general, MCL lesions are located on, or connected to the nasal or buccal mucosa, while DCL lesions are multiple and often present on different body parts. *Leishmania recidivans* (LR) is a reactivated lesion on the border of a previously healed scar.

#### Data analysis

Data analysis was done in R version 3.6.1 [28]. For the baseline table, chi-square or Fisher’s exact tests were used for categorical variables and two-sample t-tests or Mann-Whitney tests for continuous variables to compare between sites.

For the treatment outcome analyses, multinomial regression was done with calculation of 95% confidence intervals (CI) based on the model. While the primary analysis was done using the PP population for day 90, additional analyses included the ITT and confirmed CL populations and also using only the index lesion. Outcome per age group and type of CL was also analyzed for day 90 using multinomial regression to test for differences between groups.

Poisson regression was used with adherence as a continuous variable (number of missed days) to calculate adjusted mean adherence and 95% confidence intervals. Side-effects were analyzed with logistic regression.

As an exploratory objective, we developed a multivariable prognostic prediction model to predict cure at day 90 and to predict relapse at day 180 to identify patients who could benefit more from miltefosine treatment. Multiple logistic regression was used, with predictors having a p-value < 0.1 in the bivariable model included in the multivariable model. Subsequently, variables with a p-value > 0.05 were dropped from the model by dropping the variable with the largest p-value until no more variables could be dropped.

Adjustment by site as a fixed effect with effect coding (as a covariate) was done for all analyses in which the population from both sites were grouped, to account for differences in outcomes per site.

In this analysis, each site counts equally, irrespective of the number of patients recruited.

## Results

**Fig 1:**
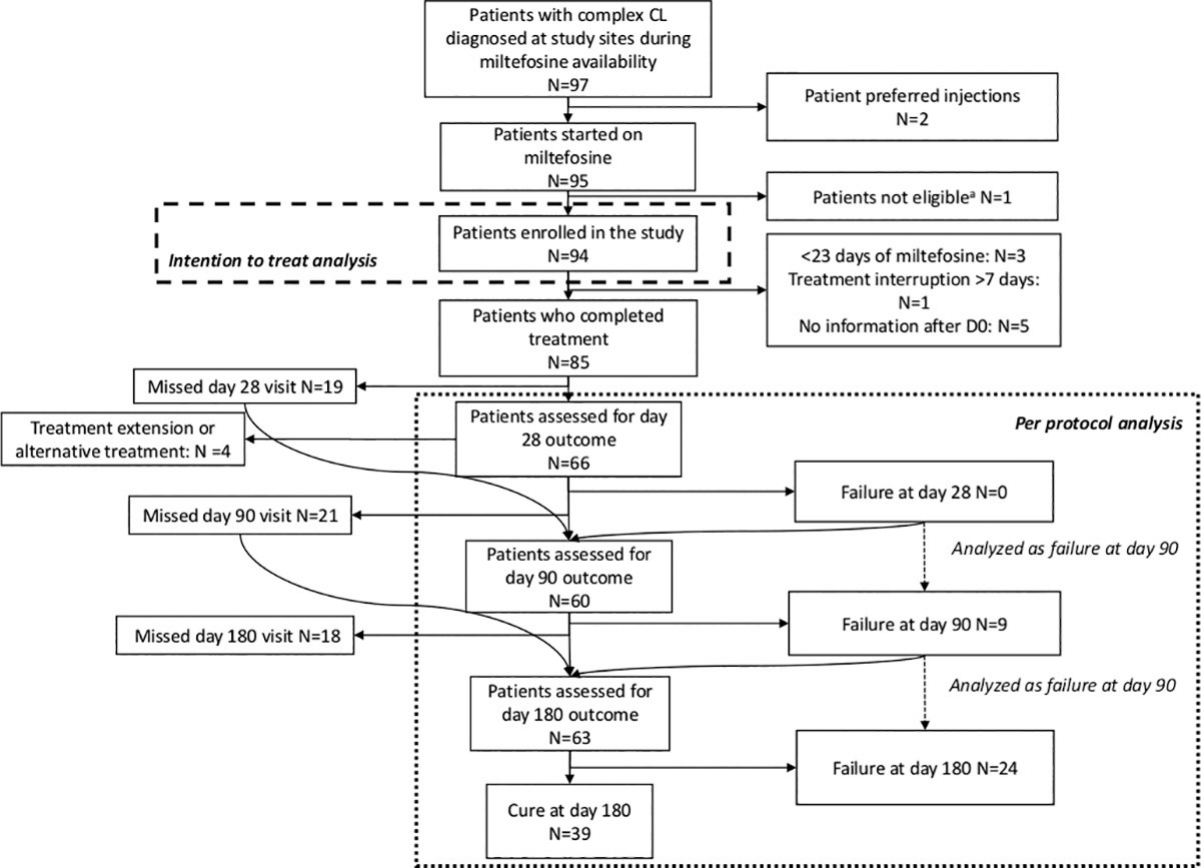
Flow chart of patients. ^a^We initially only planned to include microscopy confirmed patients, but due to the high proportion of patients treated empirically, we amended the inclusion criteria. One patient treated empirically was excluded because at the time of starting miltefosine, the amendment was not yet approved.

Amongst 97 patients screened, 94 patients were included, 32 from Gondar and 62 from Boru Meda Hospital (Fig 1). Two of the screened patients preferred to take SSG intramuscular injections rather than oral miltefosine. The patients included are described in Table 1. Most patients were male (63/94, 67.0%) and young (median age 21.5) and median lesion size was six cm. Median lesion duration was 12.0 months, but more than a quarter of patients had lesions for over two years. Almost all (86/94, 91.5%) lesions were on the face. The most common form of CL was MCL (47/94, 50.0%), followed by DCL (26/94, 27.7%), and LCL (19/94, 20.2%). Two patients (2.1%) were classified as LR. Frequent lesion features were plaques (73/94, 77.7%), erythema (49/94, 52.1%), crusts (48/94, 51.1%), swelling (38/94, 40.4%), nodules (28/94, 29.8%), papules (26/94, 27.7%), and scaly lesions (25/94, 26.6%). Ulceration was present in only 12 (12.8%) lesions. Several patients were treatment resistant, eight recruited patients came with relapse CL and around 20 percent were recruited after failing to respond to previous modern CL treatment, with six patients already having received more than 100 doses of SSG-based treatment.

Many patients (69/94, 73.4%) had used traditional treatment prior to presentation, especially in Gondar. Other differences between the sites were that in Gondar, lesion features were more often classified as erythematous, crusted, swollen, and scarred, while in Boru Meda lesions were more often scaly and exfoliative. The proportion of patients with DCL was higher in Gondar (12/32, 37.5%) than in Boru Meda (14/62, 22.6%) (Table 2).

**Table 2:** Baseline table.

Characteristic	Total (N = 94)	Gondar (n = 32)	Boru Meda (n = 62)	P^a^
Male sex, n (%)^b^	63 (67.0)	19 (59.4)	44 (71.0)	0.257
Age (years), median (IQR)	21.5 (16.0–39.0)	22.5 (16.0–40.0)	21.5 (16.3–33.8)	0.839
Duration of lesion (months), median (IQR)	12.0 (6.3–36.0)	12.0 (6.0–36.0)	12.0 (7.3–34.5)	0.813
Nr of lesions, median (IQR)	1.0 (1.0–2.0)	1.0 (1.0–3.0)	1.0 (1.0–2.0)	0.813
Size of lesion^c^ (cm), median (IQR)	6.0 (4.0–7.9)	7.5 (4.0–11.3)	5.5 (4.3–7.0)	0.813
Location of index lesion, n (%)				0.699
Face	86 (91.5)	30 (93.9)	56 (90.3)	
Arms and legs	8 (8.5)	2 (6.2)	6 (9.7)	
Type of CL, n (%)				0.340
LCL	19 (20.2)	7 (21.9)	12 (19.4)	
MCL	47 (50.0)	13 (40.6)	34 (54.8)	
DCL	26 (27.7)	12 (37.5)	14 (22.6)	
LR	2 (2.1)		2 (3.2)	
Presentation of index lesion^d^, n (%)				
Plaque	73 (77.7)	23 (71.9)	50 (80.6)	0.333
Erythematous	49 (52.1)	27 (84.4)	22 (35.5)	<0.001
Crusted	48 (51.1)	22 (68.8)	26 (41.9)	0.014
Swollen	38 (40.4)	25 (78.1)	13 (21.0)	<0.001
Nodular	28 (29.8)	7 (21.9)	21 (33.9)	0.228
Papular	26 (27.7)	8 (25.0)	18 (29.0)	0.679
Scaly	25 (26.6)	4 (12.5)	21 (33.9)	0.026
Ulcerated	12 (12.8)	3 (9.4)	9 (14.5)	0.745
Superinfected	10 (10.6)	7 (21.9)	10 (16.1)	0.493
Microscopy result, n (%)				
Positive^e^	46 (53.5)	22 (71.0)	24 (43.6)	0.015
Negative	40 (46.5)	9 (29.0)	31 (56.4)	
PCR result, n (%)				0.346
Positive^e^	79 (91.9)	28 (93.3)	51 (91.1)	
Negative	1 (1.2)	1 (3.3)	0	
Invalid	6 (7.0)	1 (3.3)	5 (8.9)	
HIV coinfection^e^, n (%)	2 (2.5)	1 (5.6)	1 (1.6)	0.406
Relapse, n (%)	8 (8.5)	2 (6.2)	6 (9.7)	0.712
Use of prior traditional treatment	69 (73.4)	28 (87.5)	41 (66.1)	0.026
Herbal^f^	59 (62.8)	23 (71.9)	36 (58.1)	0.189
Use of prior modern treatment	19 (20.2)	8 (25.0)	11 (17.7)	0.406
SSG IL	2	-	2	
Cryotherapy	1	1	-	
SSG IM/IV	10^g^	7	3	
SSG IM/IV + allopurinol	7^h^	-	7	
AmBisome	1^i^	1	-	
SSG + paromomycin	1^j^	2	-	

LCL: localized cutaneous leishmaniasis, LR: *leishmania* recidivans, MCL: mucocutaneous leishmaniasis, DCL: diffuse cutaneous leishmaniasis, IQR: interquartile range, PCR: polymerase chain reaction, HIV: human immunodeficiency virus, SSG: sodium stibogluconate, IL: intralesional, IM: intramuscular, IV: intravenous.

^a^P-values of χ2,Fisher exact test, two-sample t-test or Mann-Whitney test to compare between sites.

^b^All percentages are column percentages.

^c^By largest diameter.

^d^Lesions can have multiple presentations, therefore the sum of the different categories can be larger than the whole. Additional presentations were exfoliation (n = 18), hyperpigmentation (n = 17), macrochelia (n = 16), and scarring (n = 10)

^e^% are calculated excluding missing values (8 microscopy results, 12 PCR results, 15 HIV results which were not done).

^f^ Other treatments included holy water (n = 7), heat (n = 2), ash (n = 2), rubbing with a cross (n = 2), (holy) soil/mud (n = 3), and insects (n = 1).

^g^3 patients did not complete a whole cycle due to side-effects (n = 2) and non-response (n = 1). The other patients took 1 (n = 2), 2 (n = 2), 3 (n = 1), 5 (n = 1), or 6 (n = 1) 28–43 day cycles of SSG.

^h^Patients took 1 (n = 2), 2(n = 2), 3(n = 2), 4(n = 1), or 6(n = 1) cycles of SSG + allopurinol.

^i^1 patient was given a course of AmBisome after non-response to SSG IM/IV.

^j^1 patient was given 2 cycles of SSG + paromomycin of 63 and 21 days after failure to SSG and SSG (44d)+ miltefosine (48d).

For eight patients no skin slit sample was taken at day 0, of which two were positive previously, and two at the follow-up visit. In 46/86 (53.5%) samples *Leishmania* was confirmed using microscopy. Four slides (2 microscopy positive) were not available for PCR; 79/86 (91.9%) PCRs were positive, 1 (1.2%) negative, and 6 (7.0%) invalid. For ten patients CL was never confirmed. All five patient samples from Boru Meda selected for species typing were confirmed to be *L*. *aethiopica* infections (see S1 Fig).

Adherence to the treatment was good, with an average of 1.2 missed days when using a conservative approach (Table A in S1 Table). Side-effects were common but mild; 58.7% (50/89) of the patients experienced side-effects with nausea (33/89, 40.0%), vomiting (27/89, 33.6%) and abdominal pain (6/89, 7.4%) being most common (Table B in S1 Table). Three patients stopped miltefosine due to side-effects (at day 5, 14, and 19 respectively), one patient refused to take redosing due to vomiting, and one patient interrupted treatment for two months due to repeated vomiting and self-reported drowsiness.

At day 28, all 66 patients who came (Fig 1) showed response to the drug. Outcomes are shown in Table 3. In Boru Meda, 12/50 (24.0%) patients were cured, while in Gondar 0/16 (0%) were assessed as cured, with an adjusted overall estimate of 13.6% cured. Similarly, 35/50 (70.0%) of patients in Boru Meda and 11/16 (68.8%) in Gondar showed good response. In Boru Meda, 32/44 (72.7%) and 35/48 (72.9%) of patients were cured at day 90 and 180 respectively and 6/48 (12.5%) had relapsed by day 180, while this was 2/16 (12.5%) and 4/15 (26.7%) in Gondar for those timepoints with 9/15 (60.0%) relapse at day 180.

**Table 3:** Miltefosine treatment outcomes.

**Day 28**						
	**Overall** **N = 66**^a^^,^^b^		**Boru Meda** **N = 50**		**Gondar** **N = 16**	
**Outcome**	**n (%)**	**95% CI**	**n (%)**	**95% CI**	**n (%)**	**95% CI**
Cure	12 (13.6)		12 (24.0)	14.0–37.9	0 (0)	0–22.3
Good improvement	46 (66.0)	2.2–38.5	35 (70.0)	60.0–83.9	11 (68.8)	50.0–91.0
Partial improvement	8 (20.4)	17.6–95.9	3 (6.0)	0–19.9	5 (31.2)	12.5–53.5
No improvement	0 (0)		0 (0)	0–13.9	0 (0)	0–2.3
**Day 90**						
	**Overall** **N = 60**^a^		**Boru Meda** **N = 44**		**Gondar** **N = 16**	
	**n (%)**	**95% CI**	**n (%)**	**95% CI**	**n (%)**	**95% CI**
Cure	34 (35.7)	13.1–62.1	34 (72.7)	61.4–85.9	2 (12.5)	0–40.8
Good improvement	17 (40.9)	10.9–78.0	8 (20.5)	9.1–33.6	8 (50.0)	31.3–78.3
Partial improvement	0	-	0 (0)	0–13.2	0 (0)	0–28.3
No improvement	0	-	0 (0)	0–13.2	0 (0)	0–28.3
Relapse	9 (23.4)	3.3–60.2	3 (6.8)	0–20.0	6 (37.5)	18.8–65.8
**Day 180**						
	**Overall** **N = 63**^a^		**Boru Meda** **N = 48**		**Gondar** **N = 15**	
	**n (%)**	**95% CI**	**n (%)**	**95% CI**	**n (%)**	**95% CI**
Cure	39 (48.7)	25.2–72.6	35 (72.9)	62.5–85.8	4 (26.7)	6.7–52.7
Good improvement	9 (19.0)	3.1–54.1	7 (14.6)	4.2–27.5	2 (13.3)	0–39.4
Partial improvement	0	-	0 (0)	0–12.9	0 (0)	0–26.1
No improvement	0	-	0 (0)	0–12.9	0 (0)	0–26.1
Relapse	15 (32.3)	11.8–61.0	6 (12.5)	2.0–25.4	9 (60.0)	40.0–86.1

CI: Confidence interval. This table shows the per protocol analysis.

^a^Proportions and 95% confidence intervals shown here are adjusted by site.

^b^Adjusted proportions and confidence intervals were calculated for day 28 by adding one cured, one good improvement and one partial improvement dummy patient to Gondar to obtain estimates which could otherwise not be obtained due to sparse data.

**Fig 2:**
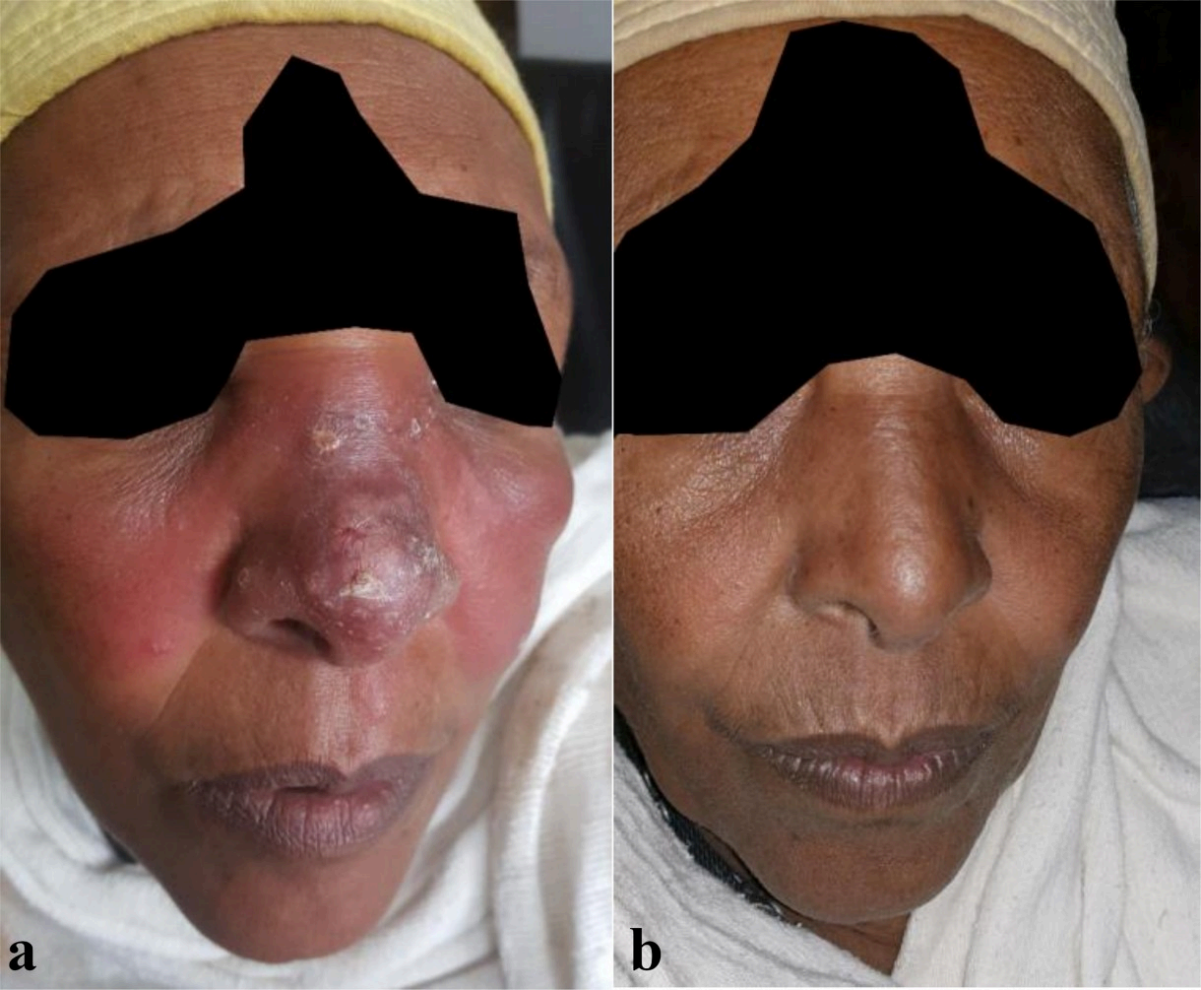
Patient with large lesion of 12 months duration classified as DCL on the nose and cheeks with features of erythema, swelling, and crusted plaques on the nose. (A) Lesion before treatment. (B) The lesion healed without residual scar at day 180.

**Fig 3:**
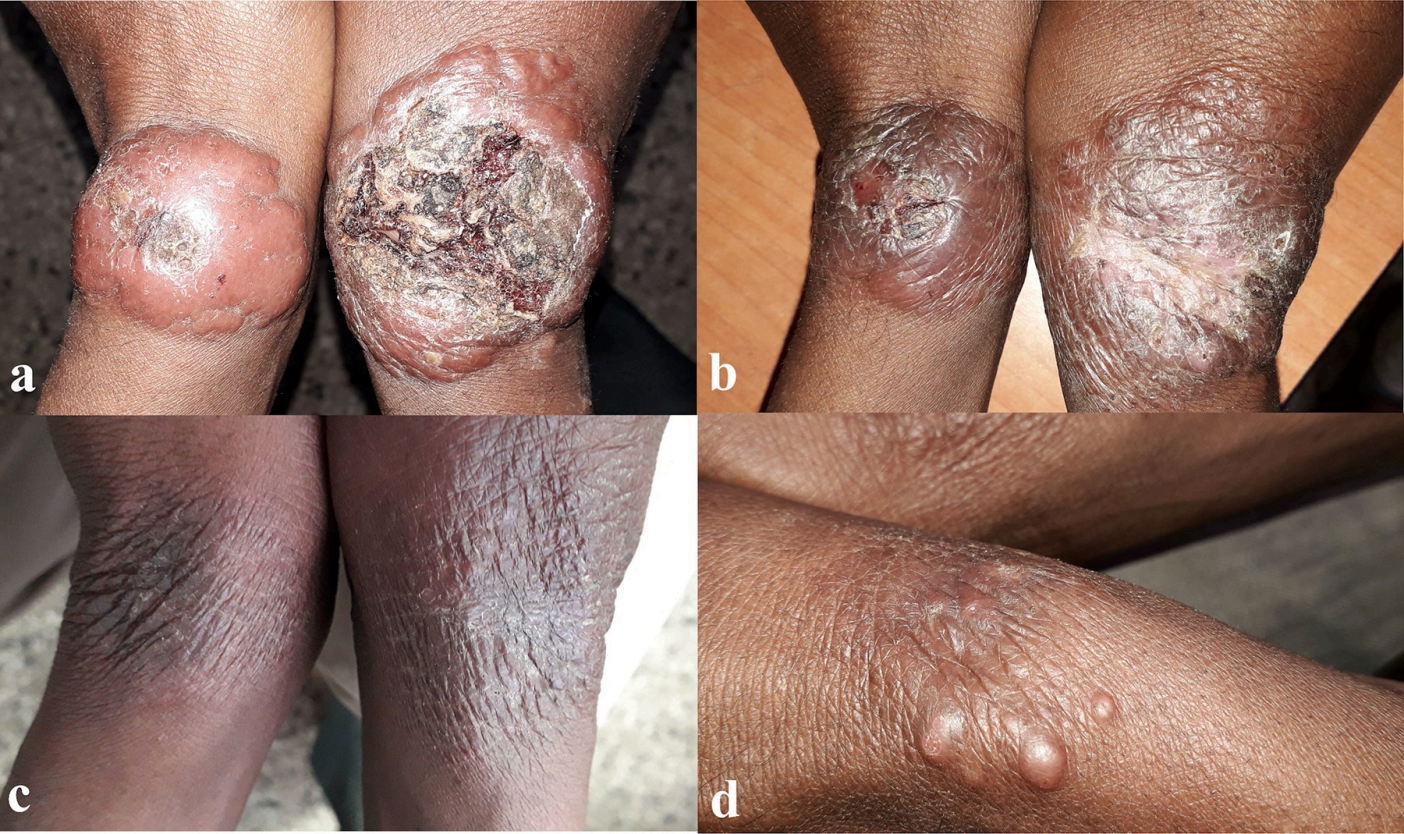
Patient with two large symmetric lesions classified as LCL on both wrists of nine months duration. Lesions show nodular erythema with central ulceration and superinfection at day 0 (A). The lesion showed progressive improvement at day 28 (B) and day 90 (C) with almost complete flattening and reepithelization. At day 180, new nodules appeared on the site of the previous lesion, indicating relapse (D).

Overall pooled estimates adjusted by site showed that 48.7% (39/63) patients were cured at day 180, 19.0% (9/63) showed good improvement without complete cure, and 32.3% (15/63) relapsed (Table 3). Relapse was most often diagnosed day 90 (n = 7) and day 180 (n = 6), but also occurred at day 60 already (n = 2). Patients who developed relapse are described in detail in Table C in S1 Table. Figs 2 and 3 provide examples of a patient with excellent response or relapse respectively.

Results from the sensitivity analyses (Table 4) showed that the proportion of patients with cure was only 19.7% (35/94) for day 90 for ITT while 24.4% (17/94) had good response. Outcomes from confirmed cases only (also see Table D in S1 Table) were similar to the main analysis with 37.1% (32/56) patients cured at day 90 whereas outcomes for the index lesion alone were slightly better, with 50.7% (37/60) of the index lesions cured at day 90, and 32.0% (15/60) of the lesions having good improvement.

**Table 4:** Sensitivity analyses for day 90 outcomes for both study sites combined.

	ITT^a^ N = 94		Confirmed only (PP) N = 56		Index only (PP) N = 60	
	n (%)	95% CI	n (%)	95% CI	n (%)	95% CI
Cure	35 (19.3)	8.3–34.0	32 (37.1)	14.4–63.8	37 (50.7)	29.5–72.9
Good improvement	18 (25.2)	4.6–59.6	15 (39.1)	9.3–75.8	15 (32.0)	11.7–58.6
Partial improvement	4 (6.2)	0.6–21.4	0	-	0	
No improvement	28 (35.0)	8.9–72.2	0	-	0	
Relapse	9 (14.3)	1.9–40.5	9 (23.9)	4.5–61.7	10 (17.4)	4.5–40.7

CI: confidence interval, ITT: Intention to treat, PP: per protocol.

All proportions and 95% Confidence intervals shown here are adjusted by site.

^a^Dummy patients were added for every outcome level for Boru Meda site to obtain estimates which could otherwise not be ontained due to sparse data.

There were several patients who were excluded from the PP analysis because they received additional treatment. Three patients received miltefosine treatment extension of 15 days because the clinician feared for relapse after finishing the standard 28 days of treatment (two of these patients had extensive DCL), but all three still showed relapse at day 180. Outcomes of patients who did not comply to the 28 days of treatment (including ones with treatment extension) are shown in Table E in S1 Table. Outcomes of patients who were not parasitologically confirmed to be CL are shown in Table F in S1 Table.

Outcomes per lesion type were not significantly different (Table 5), although relapse was seen in more LCL patients (5/12, 48.4%) compared to MCL (1/29, 9.7%) and DCL (3/17, 23.6%). In Boru Meda, cure rates for DCL (10/11, 90.0%) and MCL (19/24, 79.2%) were significantly (*p = 0*.*005*) better than for LCL patients (2/7, 28.6%) (Table G in S1 Table); there was no significant difference for Gondar (*p = 0*.*054*, Table H in S1 Table). Age below *vs* above 18 years had significantly different outcomes (*p = 0*.*035*), with less children achieving cure at day 90 (Table I in S1 Table).

**Table 5:** Treatment outcome at day 90 by leishmaniasis type.

	LCL^a^^.^^b^ N = 12		MCL N = 29		DCL N = 17		
		95% CI		95% CI		95% CI	P
Cure	4 (18.7)	3.0–49.6	19 (41.7)	12.7–76.4	10 (45.1)	15.9–73.4	0.100
Good improvement	3 (32.8)	2.6–83.7	9 (48.7)	12.3–84.1	4 (31.4)	4.7–72.2	
Partial Improvement	0		0		0		
No improvement	0		0		0		
Relapse	5 (48.4)	6.0–91.9	1 (9.7)	0.4–41.6	3 (23.6)	2.8–66.0	

CI: confidence interval, DCL: diffuse cutaneous leishmaniasis, LCL: localized cutaneous leishmaniasis, MCL: mucocutaneous leishmaniasis.

^a^All proportions shown here are adjusted by site

^b^Patients with leishmania recidivans are not shown in this table, as there were only two patients from Boru Meda classified as *leishmania recidivans*

For predictors of cure at day 90 (Table J in S1 Table), an increase in lesion size was significantly associated with a lower chance of cure (OR 0.80, 95% CI 0.61–0.99, *p = 0*.*044)*. Type of lesion was shown to be predictive of relapse (*p = 0*.*049*) at day 180, with MCL (OR 0.11, 95% CI 0.01–0.66) having lower odds of relapse compared to LCL (Table H in S1 Table). DCL response was not significantly different from LCL (OR 0.46, 95% CI 0.07–2.48).

## Discussion

In this study we provide the first evidence on the use of miltefosine for treating CL patients in Ethiopia. The reported adherence of miltefosine was very good, but since this was reported by patients (and through patient-filled adherence logs), it is possible that these results are an overestimation of the true adherence. Side-effects were common but tolerable. Most patients showed good improvement (66.0%) or cure (13.6%) 28 days after starting miltefosine, including patients who failed and relapsed after taking several cycles of systemic SSG. However, only around half the patients were cured at day 180 in pooled analysis from both study sites, and a substantial proportion of patients relapsed. Nevertheless, there seem to be subgroups of patients in which miltefosine works well; outcomes were better in patients with MCL and were remarkably better in Boru Meda with 72.7% cure at day 180 compared to 26.7% in Gondar.

The average cure rate needs to be seen in context of our severe and hard to treat CL lesions and lack of better treatment options. Our patients mostly had longstanding, large lesions on the face and there was a high proportion of patients with DCL and MCL and previous unsuccessful treatment. However, this represents the kind of patients requiring (systemic) care at CL treatment sites in Ethiopia. In contrast, most evidence on miltefosine comes from clinical trials that include only relatively mild cases with recent single ulcerative lesions without mucosal involvement and no (recent) previous treatment [19,29,30]. It remains to be studied how effective miltefosine would be in more recent cases in Ethiopia, for example those identified during active case finding.

Most patients included in our study would normally be treated with systemic SSG, which is usually extended beyond an initial one-month treatment cycle. In our study sites, the majority of CL patients in need of systemic treatment are treated for several months, and all clinicians indicated that initial response to SSG after one month is usually less favorable compared to miltefosine, and most DCL patients never reach cure despite months of SSG treatment. Therefore, patients who show good response without reaching cure are generally considered to have a positive outcome, while for analysis of this paper, we used more strict criteria according to international recommendations. If we would consider good outcome as positive, then 58/66 (87.9%), 50/59 (84.7%) and 44/59 (74.6%) patients would be considered as positive outcomes for day 28, 90 and 180 respectively, which looks much more encouraging. We also analyzed outcomes per patient, while some papers look only at the index lesion to determine outcome assessment [26,27].

However, since we did not have a control arm it is hard to determine how miltefosine really compares to currently available treatments. There are no hospital-based studies from Ethiopia focusing on CL which allow for a valid comparison to our study population, as the only two studies that looked at long term outcomes for a single regimen of antimonials are on less-severe patient populations [2,31]. A retrospective study from Gondar using a similar population as ours but without proper follow-up showed that only 25% of patients with known outcomes were cured after one month of systemic SSG [7]. Comparative trials focusing on safety, efficacy, and patient preference may shed more light on whether miltefosine is preferable to using antimonials.

The initial good response of miltefosine indicates good antileishmanial activity, but the response does not seem to be maintained in a subgroup of patients. Similar results were observed in other case series in Latin America, which showed an initial good response in (mostly DCL) patients followed by relapse after treatment cessation. Extending miltefosine from four to six weeks did not increase cure rates for CL [32], and also does not seem to prevent relapse [33–35]; as one study showed that 15/16 DCL patients eventually relapsed, despite treatment of at least 75 days. For three of our study patients, the physician also decided to extend to a total of six weeks of miltefosine, but all of these patients still relapsed at day 180.

Rather, we hypothesize that the non-sustained response to miltefosine suggests a lack of protective effector immunity or long-term memory immunity after drug absence to combat the recrudescence of residual hard-to-reach parasites. Therefore, strengthening the correct protective immunity development by combining miltefosine treatment with another immunomodulatory drug could improve outcomes in order to achieve stable and sterile cure and also decrease chance of developing resistance to miltefosine. Similar calls were raised by other groups [34,35], and a similar approach is being used for VL, where patients are only given miltefosine as combination therapy in East Africa.

To understand the striking difference between the study sites further studies are needed, but we can speculate on several potential important factors. A different setup in patient care between the sites (1 week admission vs completely outpatient treatment) could have affected adherence and loss to follow-up, there could be differences in severity of patient populations in the two sites (although not evident statistically) or differences in circulating parasites strains and their virulence, and lastly, there may have been differences among physicians doing outcome assessments, as outcome assessment is currently subjective. Given this difference between the sites, we have mainly focused on reporting the outcomes for each site separately. Such site specific outcome estimates, and the reasons causing them, are vital information for the conduct of clinical trials in the future.

Besides the small sample size and lack of a control arm, limitations of this study are a relatively high loss to follow-up (potentially related to outcome), possibility of inclusion of patients who may not truly have had CL, subjective classification and outcome assessment, and a follow-up of 180 days which may be considered short for severe CL lesions. Although we tried to adhere to recommended methodology [26] as much as possible, there are several points to be improved for future studies, which mostly relate to the need for standardization.

Consensus approved operational definitions for CL classification are highly needed, and should be context-specific rather than directly transposed from other *Leishmania* species. Immunological and parasitological studies could provide more insight in this matter. More objective measures for outcome assessment are required, especially since non-ulcerated and often diffusely swollen plaque-like lesions are hard to objectively judge based on photographs alone. Consensus methods using a combined assessment of multiple dermatologists, or standardized techniques such as a 3-dimensional scanner [36,37] could be used for outcome assessment.

Standard provision of injectable contraceptives should be provided for women of childbearing age. In our study, we counseled women extensively on the use of contraceptives, and did not enroll women who were not willing to use them or abstain from sexual interactions. None of the female patients who were ’of childbearing age’ we enrolled became pregnant during the study follow-up, but more stringent measures should be taken in future studies.

Overall, miltefosine seems promising due to its good initial response, tolerable side-effects, and its tablet form allowing outpatient treatment. However, for CL in Ethiopia, where lesions are generally severe and of long-standing duration, miltefosine monotherapy does not seem to be sufficient to achieve sustained cure in all patients. We propose to include miltefosine in future clinical trials, either as combination therapy or targeting specific subgroups. Exploration of miltefosine for treatment of less severe lesions or lesions of shorter duration both in hospital and community settings may also be worthwhile. It is vital that such trials aim to standardize procedures as much as possible.

## Supporting information

S1 STROBE checklist Checklist of items that should be included in reports of observational studies. (DOC)

S1 Fig Neighbor joining tree of ITS-1 sequences of five clinical samples from Boru Meda and other relevant *Leishmania* species. *Leishmania aethiopica*, *L*. *tropica* and *L*. *major* reference samples were derived from clinical isolates originating from Ethiopia, Egypt and Sudan respectively. The dendrogram was based on p-distances.
(TIF)

S1 Table Supporting tables. (DOCX)

S1 Text Detailed methods. (DOCX)
