# Supplementary material for: Miltefosine for the treatment of cutaneous leishmaniasis—A pilot study from Ethiopia
Source: PLoS Negl Trop Dis. 2021 May 28;15(5):e0009460. doi: 10.1371/journal.pntd.0009460 (PMC8191986; doi:10.1371/journal.pntd.0009460)
Supplement: S1 STROBE checklist — (DOC) [file pntd.0009460.s001.doc]

STROBE Statement—checklist of items that should be included in reports of observational studies

|  | Item No | Recommendation | Manuscript section and paragraph nr |
| --- | --- | --- | --- |
| **Title and abstract** | 1 | (*a*) Indicate the study’s design with a commonly used term in the title or the abstract | Abstract: methodology and principal findings |
|  |  | (*b*) Provide in the abstract an informative and balanced summary of what was done and what was found | Abstract: methodology and principal findings |
| Introduction | | |  |
| Background/rationale | 2 | Explain the scientific background and rationale for the investigation being reported | Introduction: paragraph 1-7 |
| Objectives | 3 | State specific objectives, including any prespecified hypotheses | Introduction: paragraph 7 |
| Methods | | |  |
| Study design | 4 | Present key elements of study design early in the paper | Methods, subsection "study design, population and treatment": paragraph 1 |
| Setting | 5 | Describe the setting, locations, and relevant dates, including periods of recruitment, exposure, follow-up, and data collection | Setting and locations methods, subsection "study setting": paragraph 1-3, relevant dates in methods, subsection "study design, population and recruitment": paragraph 2. |
| Participants | 6 | (*a*) *Cohort study*—Give the eligibility criteria, and the sources and methods of selection of participants. Describe methods of follow-up  *Case-control study*—Give the eligibility criteria, and the sources and methods of case ascertainment and control selection. Give the rationale for the choice of cases and controls  *Cross-sectional study*—Give the eligibility criteria, and the sources and methods of selection of participants | Eligibility criteria, source population and patient selection in methods, subsection "study design, population and recruitment": paragraph 1 |
|  |  | (*b*) *Cohort study*—For matched studies, give matching criteria and number of exposed and unexposed  *Case-control study*—For matched studies, give matching criteria and the number of controls per case | - |
| Variables | 7 | Clearly define all outcomes, exposures, predictors, potential confounders, and effect modifiers. Give diagnostic criteria, if applicable | Outcome definitions and other variable definitions in methods, subsection "data collection and analysis: definitions": paragraph 1-2  Diagnostic variables in methods, subsection "CL confirmation": and S1 methods. |
| Data sources/ measurement | 8* | For each variable of interest, give sources of data and details of methods of assessment (measurement). Describe comparability of assessment methods if there is more than one group | Outcome assessment in methods, subsection "data collection and analysis: definitions" |
| Bias | 9 | Describe any efforts to address potential sources of bias | Methods, "data collection and analysis: data analysis" |
| Study size | 10 | Explain how the study size was arrived at | Methods, subsection "study design, population and recruitment": paragraph 2 |
| Quantitative variables | 11 | Explain how quantitative variables were handled in the analyses. If applicable, describe which groupings were chosen and why | Quantitative information was not grouped, see Table 2. |
| Statistical methods | 12 | (*a*) Describe all statistical methods, including those used to control for confounding | Methods, subsection , "data collection and analysis: data analysis": paragraph 1-5 |
|  |  | (*b*) Describe any methods used to examine subgroups and interactions | Methods, subsection , "data collection and analysis: data analysis": paragraph 2 |
|  |  | (*c*) Explain how missing data were addressed | Methods, "data collection and analysis: analysis populations", and "data collection and analysis: definitions": paragraph 2 as well as S1 supplementary methods: additional information on outcome assessment: paragraph 1-2. |
|  |  | (*d*) *Cohort study*—If applicable, explain how loss to follow-up was addressed  *Case-control study*—If applicable, explain how matching of cases and controls was addressed  *Cross-sectional study*—If applicable, describe analytical methods taking account of sampling strategy | Methods, "data collection and analysis: analysis populations", and "data collection and analysis: definitions": paragraph 2 as well as S1 methods: additional information on outcome assessment: paragraph 1-2. |
|  |  | (*e*) Describe any sensitivity analyses | Methods, "data collection and analysis: analysis populations" |

Continued on next page

| Results | | |  |
| --- | --- | --- | --- |
| Participants | 13* | (a) Report numbers of individuals at each stage of study—eg numbers potentially eligible, examined for eligibility, confirmed eligible, included in the study, completing follow-up, and analysed | Fig 1. |
|  |  | (b) Give reasons for non-participation at each stage | Fig 1. |
|  |  | (c) Consider use of a flow diagram | Fig 1. |
| Descriptive data | 14* | (a) Give characteristics of study participants (eg demographic, clinical, social) and information on exposures and potential confounders | Table 2 |
|  |  | (b) Indicate number of participants with missing data for each variable of interest | Table 2 and Table 3 |
|  |  | (c) *Cohort study*—Summarise follow-up time (eg, average and total amount) | The total number of patients who visited each visit is shown in Table 3. |
| Outcome data | 15* | *Cohort study*—Report numbers of outcome events or summary measures over time | Table 3. |
|  |  | *Case-control study—*Report numbers in each exposure category, or summary measures of exposure | *-* |
|  |  | *Cross-sectional study—*Report numbers of outcome events or summary measures | *-* |
| Main results | 16 | (*a*) Give unadjusted estimates and, if applicable, confounder-adjusted estimates and their precision (eg, 95% confidence interval). Make clear which confounders were adjusted for and why they were included | Adjusted overall estimates are shown in Table 4, unadjusted estimates are shown in Table 3. |
|  |  | (*b*) Report category boundaries when continuous variables were categorized | NA |
|  |  | (*c*) If relevant, consider translating estimates of relative risk into absolute risk for a meaningful time period | NA |
| Other analyses | 17 | Report other analyses done—eg analyses of subgroups and interactions, and sensitivity analyses | Sensitivity analyses: S1 Tables 4, subgroup analyses:S1 Tables 7-10 |
| Discussion | | |  |
| Key results | 18 | Summarise key results with reference to study objectives | Discussion paragraph 1. |
| Limitations | 19 | Discuss limitations of the study, taking into account sources of potential bias or imprecision. Discuss both direction and magnitude of any potential bias | Bias for adherence: paragraph 1, limitations in paragraph 4 & paragraph 8. |
| Interpretation | 20 | Give a cautious overall interpretation of results considering objectives, limitations, multiplicity of analyses, results from similar studies, and other relevant evidence | Paragraph 2,3,5,6 and 11. |
| Generalisability | 21 | Discuss the generalisability (external validity) of the study results | Paragraph 2 |
| Other information | | |  |
| Funding | 22 | Give the source of funding and the role of the funders for the present study and, if applicable, for the original study on which the present article is based | Financial disclosure statement |

*Give information separately for cases and controls in case-control studies and, if applicable, for exposed and unexposed groups in cohort and cross-sectional studies.

**Note:** An Explanation and Elaboration article discusses each checklist item and gives methodological background and published examples of transparent reporting. The STROBE checklist is best used in conjunction with this article (freely available on the Web sites of PLoS Medicine at http://www.plosmedicine.org/, Annals of Internal Medicine at http://www.annals.org/, and Epidemiology at http://www.epidem.com/). Information on the STROBE Initiative is available at www.strobe-statement.org.
