# Supplementary figures and images for: Miltefosine for the treatment of cutaneous leishmaniasis—A pilot study from Ethiopia
Source: PLoS Negl Trop Dis. 2021 May 28;15(5):e0009460. doi: 10.1371/journal.pntd.0009460 (PMC8191986; doi:10.1371/journal.pntd.0009460)

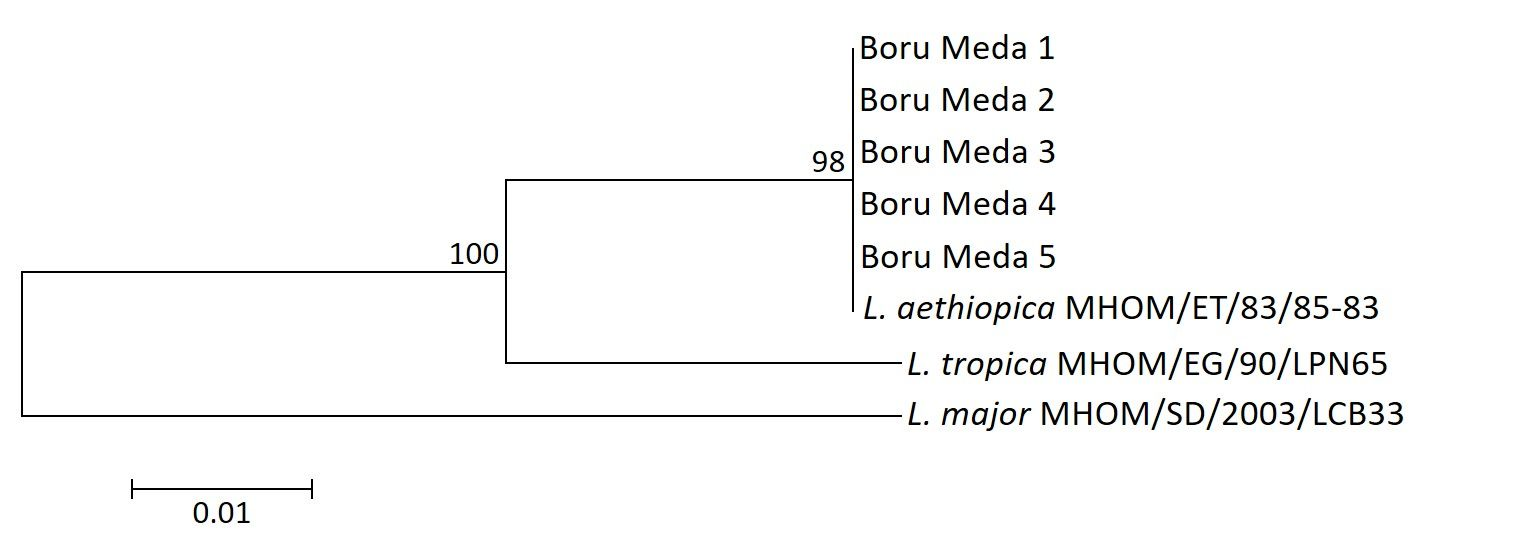

Supplement: S1 Fig — Leishmania aethiopica, L. tropica and L. major reference samples were derived from clinical isolates originating from Ethiopia, Egypt and Sudan respectively. The dendrogram was based on p-distances. (TIF) [file pntd.0009460.s002.tif]
