# Supplementary material for: Miltefosine for the treatment of cutaneous leishmaniasis—A pilot study from Ethiopia
Source: PLoS Negl Trop Dis. 2021 May 28;15(5):e0009460. doi: 10.1371/journal.pntd.0009460 (PMC8191986; doi:10.1371/journal.pntd.0009460)
Supplement: S1 Table — (DOCX) [file pntd.0009460.s003.docx]

**S1 Tables**

**A.** Adherence to 28 days of miltefosine treatment

|  | **Worst-case scenario** | | **Missing excluded** | |
| --- | --- | --- | --- | --- |
|  | N=94 | | N=89 | |
|  |  | 95% CI |  | 95% CI |
| Mean days of missed treatment | 1.2 | 0.95-1.41 | 0.6 | 0.5 – 0.8 |

CI: confidence interval.

All values are adjusted by site.

**B.** Side-effects experienced during treatment with miltefosine

|  | **N=89** | **95% CI** |
| --- | --- | --- |
| Side-effects | 50 (58.7) | 47.7 – 69.1 |
| Nausea | 33 (40.0) | 29.2 – 50.5 |
| Vomiting | 27 (33.6) | 23.7 – 45.2 |
| Abdominal pain | 6 (7.4) | 2.9 – 15.3 |
| Diarrhea | 4 (5.2) | 1.8 – 12.2 |
| Headache^a^ | 3 (4.7) | 0.1 – 12.4 |
| Weakness | 3 (3.9) | 1.0 – 10.0 |
| Fever | 0 (0) | - |

^a^2 Dummy patients were added for Boru Meda patients (1 with, 1 without headache) to get reliable estimates

CI: confidence interval.

All proportions and 95% confidence intervals are adjusted by site.

**C.** Line list of patients with relapse

| **Age** | **Sex** | **Duration of lesion (months)** | **Previous CL** | **Type of CL** | **Nr lesions** | **Size index lesion (largest x smallest diameter)** | **CL confirmed (microscopy grade)** ^a^ | **Daily doses of MF taken** | **Day 28 outcome** | **Other treatments given** | **Day 60 outcome** | **Day 90 outcome** | **Day 180 outcome** |
| --- | --- | --- | --- | --- | --- | --- | --- | --- | --- | --- | --- | --- | --- |
| 40 | M | 9 | N | LCL | 2 | 11x6 | N (0) | 28 | Partial response | No |  | Good response | Relapse |
| 15 | M | 8 | N | DCL | 3 | 7x5 | Y (+1) | 28 | Good response | No |  | Cure | Relapse |
| 15 | M | 24 | N | LCL | 2 | 10x5 | Y (+4) | 28 | Good response | No |  | Relapse | Relapse |
| 20 | M | 9 | N | LCL | 2 | 12x4 | Y (+2^b^) | 28 | Cure (still parasites) | No |  | Relapse | *Missed* |
| 6 | F | 24 | N | DCL | 3 | 11x9 | Y (0) | 28 | Good response | No |  | Relapse | Relapse |
| 30 | M | 5 | N | LCL | 1 | 4x4 | Y (+3) | 28 | Partial response | 6 weeks of MF was given between D90 and D180 | Relapse | Relapse | No response |
| 39 | M | 312 | N^c^ | DCL | 99^d^ | 3x1.5 | Y (+6) | 28 | Good response |  | Relapse (cured index lesion, new lesions) | Relapse (cured index lesion, worsening of new lesions) | *Missed* |
| 40 | F | 36 | 0 | MCL | 1 | 5x4 | Y (0) | 28 | Good response |  |  | *Missed* | Relapse (worsening existing lesion and new lesions) |
| 48 | M | 9 | 0 | MCL | 1 | 14x8 | Y (+3) | 28 | *Missed* | SSG initiated after D90- visit |  | Relapse | *Missed* |
| 16 | F | 48 | 0 | DCL | 3 | 7x7 | Y (+1) | 28 | Good response |  |  | Good response (few suspicious lesions) | Relapse (worsening existing lesion) |
| 13 | F | 6 | 0 | LCL | 1 | 3.5x2.5 | Y (+3) | 28 | Good response | Cryotherapy twice after day 90 visit |  | Relapse (new lesion) | Good response |
| 25 | M | 5 | N | DCL | 5 | 3x3 | Y (+2) | 27 | Partial response | SSG for 54 days after day 90 visit |  | Relapse | Good response |
| 11 | M | 60 | N | DCL | 1 | 16x4 | Y (0) | 28 | Good response |  |  | *Missed* | Relapse (new lesions and worsening exisiting lesion) |
| 2 | F | 6 | N | LCL | 1 | 4x3 | Y (0) | 25 | Partial response | Cryotherapy after D90 | Good response | Relapse (new lesions) | *Missed* |
| 50 | M | 11 | Y (1) | DCL | 62 | 9x6 | Y (+5) | 28 | Good response | Extension of MF with 15 days after D28 |  | Good response | Relapse (cured index lesion, reactivation peripheral lesions) |
| 24 | M | 180 | N | DCL | 99 | 110x36 | Y (+6) | 28 | Good response | Extension of MF with 15 days after day 28 visit | Good response | Relapse (new lesions) | Relapse (new lesion) |
| 16 | M | 14 | N | MCL | 1 | 11x7 | Y (+2) | 28 | Good response | 15 days of MF was given after day 28 visit | Good response | Good response | Relapse (new lesion) |

CL: cutaneous leishmaniasis, DCL: diffuse cutaneous leishmaniasis, LCL: localized cutaneous leishmaniasis, MCL: mucocutaneous leishmaniasis, MF: miltefosine, SSG: sodium stibogluconate

^a^CL confirmation either through microscopy or PCR

^b^Microscopy grade is from the slide taken at day 28, no skin slit was performed at day 0

^c^The patient had a long history with CL with several relapsing episodes without ever achieving cure. These are counted as one continuous episode

^d^When number of lesions was too many too count, 99 was used

**D.** Sensitivity analysis on confirmed CL patients only for both study sites combined, using the per protocol population

|  | **D28**  **N=63** | | **D90**  **N=56** | | **D180**  **N=58** | |
| --- | --- | --- | --- | --- | --- | --- |
|  | **n (%)** | **95% CI** | **n (%)** | **95% CI** | **n (%)** | **95% CI** |
| Cure | 12 (14.1) | 2.0 – 39.7 | 32 (37.1) | 14.4 – 63.8 | 35 (48.8) | 26.1 – 69.9 |
| Good improvement | 44 (66.9) | 18.0 – 96.1 | 15 (39.1) | 9.3 – 75.8 | 8 (18.2) | 2.8 – 50.8 |
| Partial improvement | 7 (19.0) | 1.0 – 66.9 | 0 | - | 0 |  |
| No improvement | 0 (0) |  | 0 | - | 0 |  |
| Relapse |  |  | 9 (23.9) | 4.5 – 61.7 | 15 (33.0) | 11.3 – 59.3 |

All proportions and 95% confidence intervals are adjusted by site.

**E.** Line list of patients with non-compliance to 28 days of treatment (including alternative treatments)

| **Age** | **Sex** | **Duration of lesion (months)** | **Previous CL** | **Type of CL** | **Number of lesions** | **Size index lesion (largest x smallest diameter)** | **Other info** | **Days of MF taken** | **Treatment interruption (days)** | **Reason** | **Other treatments given** | **Day 28 outcome** | **Day 60 outcome** | **Day 90 outcome** | **Day 180 outcome** |
| --- | --- | --- | --- | --- | --- | --- | --- | --- | --- | --- | --- | --- | --- | --- | --- |
| 42 | M | 24 | N | MCL | 1 | 6x4 | - | 5 |  | Stopped due to side-effects |  | *Missed* |  | *Missed* | *Missed* |
| 50 | F | 4 | N | LCL | 1 | 3x2 | Received 9 doses of SSG just before starting MF (stopped due to side-effects) | 14 |  | Stopped due to side-effects |  | *Missed* |  | Partial response | *Missed* |
| 20 | M | 72 | N | DCL | 4 (many scars) | 4x2 | No response to several cycles of SSG + allopurinol | 19 |  | Stopped due to side-effects |  | *Missed* |  | Cure | Cure |
| 45 | M | 3 | N | MCL | 1 | 5x4 | - | 24 |  | Refused to take redoses due to side-effects |  | Good response |  | Cure | *missed* |
| 2 | F | 6 | N | LCL | 1 | 4x3 | - | 25 |  | Child refused to take 3 last doses | Cryotherapy after day 90 visit | Partial | Good response | Relapse | *Missed* |
| 65 | F | 36 | Y | MCL | 3 | 10x3 | No response to 8 doses of AmBisome 4 months before starting MF | 28 | 60 | Interruption due to drowsiness, no problems at restart |  | *Missed* | - | *Missed* | Partial response |
| 50 | M | 11 | Y | DCL | 62 | 9x6 | HIV positive,  Received 9 doses (side-effects) of SSG with good improvement just before starting MF | 28 |  | Physician wanted to continue treatment | 15 days of MF after day 28 visit | Good response | - | Good response | Relapse |
| 30 | M | 5 | N | LCL | 1 | 4x4 |  | 28 |  | Rescue treatment after relapse | 42 days of MF after day 90 visit | Partial response | Relapse | Relapse | No response |
| 16 | M | 14 | N | MCL | 1 | 11x7 |  | 28 |  | Physician wanted to continue treatment | 15 days of MF after day 28 visit | Good response | Good response | Good response | Relapse |
| 48 | M | 9 | N | MCL | 1 | 14x8 |  | 28 |  | Rescue treatment after relapse | Started on SSG after day 90 visit | *Missed* | - | Relapse | *Missed* |
| 13 | F | 6 | N | LCL | 1 | 3.5x2.5 | Previous failure to cryotherapy (3 weeks prior to MF) | 28 |  | Rescue treatment after relapse | Cryotherapy twice after day 90 visit | Good response |  | Relapse | Good response |
| 25 | M | 5 | N | DCL | 5 | 3x3 |  | 27 |  | Rescue treatment after relapse | 28 days of SSG after day 90 visit | Partial response |  | Relapse | Good response |
| 16 | F | 6 | N | MCL | 1 | 9x5 |  | 28 |  | Physician wanted to restart treatment | 43 days of SSG after day 60 visit | Partial response | Partial response | Good response | *Missed* |
| 13 | M | 60 | N | DCL | 3 | 10x8 |  | 28 | 7 days | Interruption due to fever (deemed unrelated) after 24 days |  | Good response |  | Cure | Cure |
| 52 | M |  | N (84) | MCL | N | N | SSG + allopurinol for 28 days in 2017, 45 days in 2019 (2 months before MF) without response | 28 | 5 days | Interruption due to severe bilateral endophthalmitis (deemed unrelated) |  | Good response | *Missed* | *Missed* | Good response |

CL: cutaneous leishmaniasis, DCL: diffuse cutaneous leishmaniasis, LCL: localized cutaneous leishmaniasis, MCL: mucocutaneous leishmaniasis, MF: miltefosine, SSG: sodium stibogluconate.

| **Age** | **Sex** | **Previous CL** | **Duration (months)** | **Type of CL** | **Number of lesions** | **Size (largest x smallest diameter in cm(** | **Daily MF doses taken** | **Microscopy** | **PCR** | **Day 28 outcome** | **Other treatments given** | **Day 60 outcome** | **Day 90 outcome** | **Day 180 outcome** |
| --- | --- | --- | --- | --- | --- | --- | --- | --- | --- | --- | --- | --- | --- | --- |
| 30 | F | N | 8 | MCL | 1 | 6x5 | *NA* | Negative | Negative | *Missed* |  |  | *Missed* | *Missed* |
| 16 | M | N | 120 | MCL | 1 (2 scars) | 10x8 | 28 | Negative | *Invalid* | Good response |  |  | Good response | *Missed* |
| 70 | M | N | 24 | DCL | 3 | 8x4 | 28 | *Not done* | *Not done* | *Missed* |  | Good response | *Missed* | Cure |
| 60 | M | N | 12 | Leishmania recidivans | 2 (around scar) | 4x2 | 28 | *Not done* | *Invalid* | Cure |  |  | Cure | *Missed* |
| 19 | M | N | 84 | DCL | 3 | 10x7 | 28 | Negative | *Invalid* | Partial improvement |  |  | *Missed* | *Missed* |
| 24 | F | N | 12 | MCL | 1 | 5x3 | 28 | *Not done* | *Invalid* | Good response |  |  | Good response | Good response |
| 22 | M | N | 4 | LCL | 1 | 4x2 | *NA* | *Negative* | *Not done* | *Missed* |  |  | *Missed* |  |
| 60 | F | N | 24 | MCL | 1 | 6x4 | 28 | Negative | *invalid* | Good response |  |  | Cure | Cure (late d180) |
| 26 | M | N | 8 | LCL | 1 | 8x3 | 28 | *Negative* | *Not done* | *Missed* |  |  | *Missed* | Cure (early day 180) |
| 8 | F | Y | 5 | MCL | 2 | 5x2 | *NA* | *Not done* | *Not done* | *Missed* |  |  | *Missed* | *Missed* |

**F.** Line list of patients with unconfirmed cutaneous leishmaniasis

CL: cutaneous leishmaniasis, DCL: diffuse cutaneous leishmaniasis, LCL: localized cutaneous leishmaniasis, MCL: mucocutaneous leishmaniasis, NA: not available.

**G.** Treatment outcome at day 90 by type for Boru Meda hospital

|  | **LCL**  **N=7** | | **MCL**  **N=24** | | **DCL**  **N=11** | |  |
| --- | --- | --- | --- | --- | --- | --- | --- |
|  |  | **95% CI** |  | **95% CI** |  | **95% CI** | **P** |
| Cure | 2 (28.6) | 0–68.4 | 19 (79.2) | 66.7–95.7 | 10 (90.9) | 81.8–100 | 0.005 |
| Good improvement | 3 (42.9) | 14.3–82.6 | 5 (20.8) | 8.3–37.4 | 0 (0) | 0–15.1 |  |
| Partial improvement | 0 (0) | 0–39.8 | 0 (0) | 0–16.5 | 0 (0) | 0–15.1 |  |
| No improvement | 0 (0) | 0–39.8 | 0 (0) | 0–16.5 | 0 (0) | 0–15.1 |  |
| Relapse | 2 (28.6) | 0–68.4 | 0 (0) | 0–16.6 | 1 (9.1) | 0–24.2 |  |

CI: confidence interval, CL: cutaneous leishmaniasis, DCL: diffuse cutaneous leishmaniasis, LCL: localized cutaneous leishmaniasis, MCL: mucocutaneous leishmaniasis.

^a^Patients with leishmania recidivans are not shown in this table, as there were only 2 patients.

**H.** Treatment outcome at day 90 by type for University of Gondar

|  | **LCL**  **N=5** | | **MCL**  **N=5** | | **DCL**  **N=6** | |  |
| --- | --- | --- | --- | --- | --- | --- | --- |
|  |  | **95% CI** |  | **95% CI** |  | **95% CI** | **P** |
| Cure | 2 (40) | 20.0–91.2 | 0 (0) | 0–30.5 | 0 (0) | 0–44.8 | 0.054 |
| Good improvement | 0 | 0–51.1 | 4 (80.0) | 60.0–100 | 4 (66.7) | 50.0–100 |  |
| Partial improvement | 0 | 0–51.1 | 0 (0) | 0–30.5 | 0 (0) | 0–44.8 |  |
| No improvement | 0 | 0–51.1 | 0 (0) | 0–30.5 | 0 (0) | 0–44.8 |  |
| Relapse | 3 (60) | 0.4–100 | 1 (20.0) | 0–50.5 | 2 (33.3) | 167–78.2 |  |

CI: confidence interval, CL: cutaneous leishmaniasis, DCL: diffuse cutaneous leishmaniasis, LCL: localized cutaneous leishmaniasis, MCL: mucocutaneous leishmaniasis.

|  | **<18**  **N=20** | | **>18**  **N=40** | |  |
| --- | --- | --- | --- | --- | --- |
|  |  | **95% CI** |  | **95% CI** | **P** |
| Cure | 7 (16.1) | 2.9–42.7 | 27 (48.3) | 21.1–77.1 | 0.035 |
| Good improvement | 9 (53.5) | 9.2–92.5 | 8 (32.4) | 7.2–67.1 |  |
| Partial improvement | 0 |  | 0 |  |  |
| No improvement | 0 |  | 0 |  |  |
| Relapse | 4 (30.4) | 2.4–81.9 | 5 (19.4) | 3.2–53.0 |  |

**I**. Treatment outcome at day 90 by age for both sites combined

All proportions shown here are adjusted by site.

**J.** Predictors for cure at day 90

|  | **Total** | **Cure** | **No cure** | **Bivariate** | | | **Multivariable** | | |
| --- | --- | --- | --- | --- | --- | --- | --- | --- | --- |
| **Predictors** | **N=60** | **N=34** | **N=26** | **OR** | **95% CI** | **P value** | **aOR** | **95% CI** | **P- value** |
| Age (per year) |  |  |  | 1.04 | 0.99–1.10 | 0.101 |  |  |  |
| Sex |  |  |  |  |  |  |  |  |  |
| Male | 39 | 25 | 14 | 1 |  | 0.285 |  |  |  |
| Female | 20 | 9 | 12 | 0.39 | 0.05 – 2.08 |  |  |  |  |
| Duration of lesion (per month) |  |  |  | 1.00 | 0.98 – 1.02 | 0.903 |  |  |  |
| Previous CL | 5 | 4 | 1 | 1.57 | 0.20 – 32.57 | 0.690 |  |  |  |
| Type of CL |  |  |  |  |  |  |  |  |  |
| LCL | 12 | 4 | 8 | 1 |  | 0.397 |  |  |  |
| MCL | 29 | 19 | 10 | 2.82 | 0.55 – 15.35 |  |  |  |  |
| DCL^a^ | 19 | 12 | 7 | 3.02 | 0.52 – 19.75 |  |  |  |  |
| Number of lesions |  |  |  | 0.98 | NA - 1.04 | 0.653 |  |  |  |
| Size of lesion (cm) |  |  |  | 0.80 | 0.61 – 0.99 | 0.044 | 0.80 | 0.61 – 0.99 | 0.044 |
| Location of lesion |  |  |  |  |  |  |  |  |  |
| Face/head | 54 | 32 | 23 | 1 |  | 0.271 |  |  |  |
| Arms | 5 | 2 | 3 | 0.32 | 0.03 – 2.57 |  |  |  |  |
| Presentation of lesion |  |  |  |  |  |  |  |  |  |
| Ulcerated | 8 | 5 | 3 | 0.85 | 0.16 – 5.43 | 0.849 |  |  |  |
| Nodular | 15 | 10 | 5 | 1.09 | 0.28 – 4.51 | 0.906 |  |  |  |
| Papular | 17 | 11 | 6 | 1.66 | 0.43 – 7.35 | 0.471 |  |  |  |
| Plaque | 47 | 30 | 17 | 2.24 | 0.45 - 11.19 | 0.315 |  |  |  |
| Scaly | 16 | 13 | 3 | 2.83 | 0.43 – 7.35 | 0.160 |  |  |  |
| Scarred | 4 | 1 | 3 | 0.27 | 0.01 – 3.99 | 0.354 |  |  |  |
| Crusted | 31 | 15 | 16 | 0.70 | 0.20 – 2.41 | 0.565 |  |  |  |
| Dry | 5 | 3 | 2 | 0.52 | 0.07 – 4.35 | 0.512 |  |  |  |
| Phymatous | 6 | 5 | 1 | 5.12 | 0.53 – 143.94 | 0.177 |  |  |  |
| Macrochelial | 13 | 9 | 4 | 3.67 | 0.72 – 29.45 | 0.123 |  |  |  |
| Swollen | 20 | 8 | 12 | 1.46 | 0.31 – 10.60 | 0.649 |  |  |  |
| Exfoliative | 11 | 7 | 4 | 0.56 | 0.13 – 2.59 | 0.443 |  |  |  |
| Erythematous | 30 | 13 | 17 | 1.11 | 0.29 – 4.83 | 0.880 |  |  |  |
| Hyperpigmented | 11 | 6 | 5 | 0.62 | 0.14 – 2.95 | 0.536 |  |  |  |
| Superinfected | 8 | 4 | 4 | 0.61 | 0.11 – 3.75 | 0.584 |  |  |  |
| Positive skin slit microscopy | 34 | 15 | 19 | 0.48 | 0.13–1.79 | 0.276 |  |  |  |
| Previous failure to CL treatment | 11 | 7 | 4 | 1.72 | 0.36 – 10.73 | 0.512 |  |  |  |
| Traditional treatment | 43 | 22 | 21 | 0.50 | 0.11 – 1.93 | 0.319 |  |  |  |
| Herbal treatment | 35 | 18 | 17 | 0.53 | 0.14–1.85 | 0.324 |  |  |  |
| Adherence (per dose missed) |  |  |  | 1.21 | 0.46–4.08 | 0.714 |  |  |  |
| No doses missed | 54 | 32 | 22 | 1 |  |  |  |  |  |
| Doses missed | 5 | 2 | 3 | 1.31 | 0.11 – 17.83 | 0.832 |  |  |  |
| Treatment interruption | 2 | 1 | 1 | 1.66 | 0.04–82.21 | 0.785 |  |  |  |
| Missed day 28 visit | 10 | 3 | 7 | 0.57 | 0.09–3.70 | 0.569 |  |  |  |

aOR: adjusted odds ratio, CI: confidence interval, cLCL: complicated localized cutaneous leishmaniasis, MCL: mucocutaneous leishmaniasis, DCL: diffuse cutaneous leishmaniasis, IQR: interquartile range, OR: Odds ratio, PCR: polymerase chain reaction, HIV: human immunodeficiency virus, SSG: sodium stibogluconate, IL: intralesional, IM: intramuscular, IV: intravenous.

**^a^**Leishmania recidivans is analyzed as DCL in this table.

**H.** Predictors of relapse at day 180

|  | **Total** | **Relapse** | **No relapse** | **Bivariate** | | | **Multivariable** | | |
| --- | --- | --- | --- | --- | --- | --- | --- | --- | --- |
| **Predictors** | **N=63** | **N=15** | **N=48** | **OR** | **95% CI** | **P value** | **aOR** | **95% CI** | **P- value** |
| Age (per year) |  |  |  | 0.96 | 0.91 – 1.00 | 0.102 |  |  |  |
| Sex |  |  |  |  |  |  |  |  |  |
| Male | 41 | 10 | 31 | 1 |  | 0.930 |  |  |  |
| Female | 22 | 5 | 17 | 0.94 | 0.23 – 3.73 |  |  |  |  |
| Duration of lesion by month |  |  |  | 1.00 | 0.99-1.02 | 0.575 |  |  |  |
| Previous CL | 5 | 0 | 5 | 0 | NA | 0.234 |  |  |  |
| Type of CL |  |  |  |  |  |  |  |  |  |
| LCL | 15 | 7 | 8 | 1 |  | 0.039 | 1 |  | 0.049 |
| MCL | 29 | 2 | 27 | 0.10 | 0.01–0.61 |  | 0.11 | 0.01-0.66 |  |
| DCL^a^ | 17 | 6 | 11 | 0.45 | 0.08–2.24 |  | 0.46 | 0.07-2.48 |  |
| Number of lesions |  |  |  | 1.02 | 0.97 – 1.21 | 0.436 |  |  |  |
| Size of lesion (cm) |  |  |  | 1.12 | 0.92 – 1.37 | 0.250 |  |  |  |
| Location of lesion |  |  |  |  |  |  |  |  |  |
| Face/head | 57 | 13 | 44 | 1 |  | 0.383 |  |  |  |
| Arms | 6 | 2 | 4 | 2.34 | 0.25 – 17.11 |  |  |  |  |
| Presentation of lesion |  |  |  |  |  |  |  |  |  |
| Ulcerated | 10 | 1 | 9 | 0.23 | 0.01 – 2.08 | 0.232 |  |  |  |
| Nodular | 16 | 4 | 12 | 1.42 | 0.29 – 6.41 | 0.654 |  |  |  |
| Papular | 19 | 5 | 14 | 0.89 | 0.19 – 3.59 | 0.870 |  |  |  |
| Plaque | 52 | 11 | 41 | 0.84 | 0.17 – 4.81 | 0.832 |  |  |  |
| Scaly | 1 | 1 | 0 | ∞ | NA | 0.301 |  |  |  |
| Scarred | 4 | 1 | 3 | 0.46 | 0.02 – 5.63 | 0.562 |  |  |  |
| Crusted | 31 | 9 | 22 | 0.95 | 0.22 – 3.81 | 0.943 |  |  |  |
| Dry | 5 | 1 | 4 | 0.85 | 0.03 – 8.41 | 0.899 |  |  |  |
| Phymatous | 5 | 1 | 4 | 0.85 | 0.03 – 7.72 | 0.841 |  |  |  |
| Macrochelial | 10 | 2 | 8 | 0.84 | 0.10 – 4.88 | 0.851 |  |  |  |
| Swollen | 23 | 7 | 16 | 0.26 | 0.01 – 1.63 | 0.165 |  |  |  |
| Exfoliative | 15 | 4 | 11 | 2.16 | 0.43 – 10.76 | 0.339 |  |  |  |
| Erythematous | 31 | 13 | 18 | 5.49 | 1.06 – 41.36 | 0.042 | 5.30 | 0.89 - 45.21 | 0.067 |
| Hyperpigmented | 13 | 2 | 11 | 0.44 | 0.05 – 2.41 | 0.366 |  |  |  |
| Superinfected | 7 | 2 | 5 | 2.07 | 0.23 – 13.98 | 0.485 |  |  |  |
| Positive skin slit microscopy | 31 | 9 | 22 | 1.04 | 0.23 – 4.55 | 0.956 |  |  |  |
| Previous failure to CL treatment | 12 | 4 | 8 | 1.50 | 0.28 – 7.09 | 0.621 |  |  |  |
| Adherence (per dose missed) |  |  |  | 1.54 | 0.40 - 12.33 | 0.552 |  |  |  |
| Treatment interruption | 2 | 0 | 2 | 0 | NA – 100 | 0.460 |  |  |  |
| Missed day 28 visit | 15 | 2 | 13 | 0.19 | 0.02 – 1.98 | 0.063 |  |  |  |

cLCL: complicated localized cutaneous leishmaniasis, MCL: mucocutaneous leishmaniasis, DCL: diffuse cutaneous leishmaniasis, PCR: polymerase chain reaction, HIV: human immunodeficiency virus.

All analyses are adjusted by site.

**^a^**Leishmania recidivans is analyzed as DCL in this table.
