# Supplementary material for: Miltefosine for the treatment of cutaneous leishmaniasis—A pilot study from Ethiopia
Source: PLoS Negl Trop Dis. 2021 May 28;15(5):e0009460. doi: 10.1371/journal.pntd.0009460 (PMC8191986; doi:10.1371/journal.pntd.0009460)
Supplement: S1 Text — (DOCX) [file pntd.0009460.s004.docx]

**S1 Methods**

Molecular tests

Microscopy slide material was scraped off from the ambiently stored slides and DNA extraction was performed with the LEV blood DNA kit (Promega, Belgium) using the automated Maxwell 16 device (Promega, Belgium). Kit lysis buffer was added, followed by proteinase K. This was vortexed and incubated at 56 °C at 400 rpm for 20 minutes. Samples were loaded into the Maxwell device according to the manufacturer's instructions (cf. DNA program). In each batch, two negative extraction controls (NEC) were included. The eluted DNA samples were collected and stored at -80 until further use.

Samples were screened for *Leishmania* minicircle kinetoplast DNA (kDNA) with an in-house developed real-time PCR (‘LC kDNA PCR’) with improved detection for *L. aethiopica* . The assay has high sensitivity for all Old World *Leishmania* species (*L. aethiopica, L. tropica, L. infantum* and *L. donovani*), except *L. major* [1]. The PCR was carried out as described by *Merdekios et. al* [1]. An HBB PCR was done on negative samples to monitor extraction efficacy and PCR inhibition. As there were no additional samples to redo the extraction in case of contamination, all samples with Ct values that were less than 3 Ct’s lower or higher than the NEC were excluded from the further analyses and reported as 'invalid'.

Five clinical samples from Boru Meda with low Ct values were selected for species typing based on the internal transcribed spacer 1 (ITS-1) fragment. PCR amplification was performed as described by *Schönian et al.* with LITSR and L5.8S primers [2]. Amplicons were visualized on a 2% agarose gel and sent to BaseClear (Leiden, The Netherlands) for sanger sequencing with the primers used for PCR amplification.

Phylogenetic analyses were carried out in MEGA 5 software version 5.05 [3]. The obtained chromatograms were edited, and consensus sequences were made. Reference sequences were included from clinical *Leishmania* isolates of relevant species *L. tropica, L. aethiopica* and *L. major* obtained from the Institute of Tropical Medicine (Antwerp, Belgium). Sequences were aligned with the Clustal W tool and trimmed to equal lengths of 245 bp [4]. A neighbor-joining tree was constructed based on p-distances and pairwise gap exclusion, supported by bootstrap values calculated from 2,000 resamplings [5].

Additional information on outcome assessment

For patients receiving alternative treatments after treatment failure has been established the outcome for subsequent visits remained that of the visit at which treatment failure was determined (for all analysis populations). Patients who were started on alternative treatments before failure (who did not qualify as failure at the time of starting alternative treatments) were interpreted as failure for subsequent visits, but in the best possibly category of failure for the visit after the one at which the alternative treatment was decided (poor response for day 90, and good response for day 180). That outcome will remain for the following visits (for ITT).

Due to civil unrest in the Gondar area and travel restrictions during the 2020 COVID-19 outbreak, follow-up was disrupted. Therefore, outcomes of patients who came late for their day 180 visit but who had the same lesion outcome as their last visit were still included for day 180 analysis (e.g. cure at day 90 and still cure after the completion of the study).

**References**

1. Merdekios B, Pareyn M, Tadesse D, Eligo N, Kassa M, Jacobs BKM, et al. Evaluation of conventional and four real-time PCR methods for the detection of Leishmania on field-collected samples in Ethiopia. Schönian G, editor. PLoS Negl Trop Dis. 2021;15: e0008903. doi:10.1371/journal.pntd.0008903

2. Schönian G, Nasereddin A, Dinse N, Schweynoch C, Schallig HDF., Presber W, et al. PCR diagnosis and characterization of Leishmania in local and imported clinical samples. Diagn Microbiol Infect Dis. 2003;47: 349–358. doi:10.1016/S0732-8893(03)00093-2

3. Tamura K, Peterson D, Peterson N, Stecher G, Nei M, Kumar S. MEGA5: Molecular Evolutionary Genetics Analysis Using Maximum Likelihood, Evolutionary Distance, and Maximum Parsimony Methods. Mol Biol Evol. 2011;28: 2731–2739.

4. Julie D. Thompson, Toby J. Gibson, Frédéric Plewniak, François Jeanmougin, Desmond G. Higgins. CLUSTAL_X Windows Interface: Flexible Strategies for Multiple Sequence Alignment Aided by Quality Analysis Tools . Nucleid Acids Res. 1997;25: 4876–4882. Available: https://academic.oup.com/nar/article/25/24/4876/1747529

5. Saitou2 N, Nei M. The neighbor-joining method: a new method for reconstructing phylogenetic trees. Mol Biol Evol. 1987;4: 406–425. doi:10.1093/oxfordjournals.molbev.a040454
